# The Safety of Fluoride Compounds and Their Effect on the Human Body—A Narrative Review

**DOI:** 10.3390/ma16031242

**Published:** 2023-01-31

**Authors:** Adam Lubojanski, Dagmara Piesiak-Panczyszyn, Wojciech Zakrzewski, Wojciech Dobrzynski, Maria Szymonowicz, Zbigniew Rybak, Bartosz Mielan, Rafal J. Wiglusz, Adam Watras, Maciej Dobrzynski

**Affiliations:** 1 Department of Pediatric Dentistry and Preclinical Dentistry, Wroclaw Medical University, Krakowska 26, 50-425 Wroclaw, Poland; 2 Department of Conservative Dentistry with Endodontics, Wroclaw Medical University, Krakowska 26, 50-425 Wroclaw, Poland; 3 Pre-Clinical Research Centre, Wroclaw Medical University, Bujwida 44, 50-345 Wroclaw, Poland; 4 Department of Dentofacial Orthopedics and Orthodontics, Division of Facial Abnormalities, Wroclaw Medical University, Krakowska 26, 50-425 Wroclaw, Poland; 5 Institute of Low Temperature and Structure Research, Polish Academy of Sciences, Okolna 2, 50-422 Wroclaw, Poland

**Keywords:** fluoride, fluorosis, acute and chronic poisoning, caries risk, caries prevention

## Abstract

Fluoride is one of the elements commonly present in the human environment. Due to its characteristics, it is very widely used in medicine, dentistry, industry or agriculture. On the other hand, its universality possesses a real threat to the human body in the form of acute and chronic poisoning. The aim of this paper is to characterize the properties of fluoride and its effects on the human body, as well as the sources of its occurrence. Particular emphasis is placed on the safety of its use and optimal dosage intake, which prevents accumulation and reduces its potential side effects. The positive effect of proper fluoride supply is widely described. In order to avoid overdose, it is best to consult a specialist to properly select the dosage.

## 1. Introduction

Fluorine (fluorum) is a cyclic element that is very widespread in nature. It is the 13th most abundant element in the Earth’s crust and is a component of many minerals, such as fluorite, cryolite, fluorapatite, and topaz. It exhibits strong poisonous properties, and due to its high electronegativity and activity, it reacts with almost all elements, noble gases, and organic and inorganic compounds. For this reason, in a natural state it does not exist in an elemental form but in the form of compounds. In the air, the main form in which it can be found is hydrogen fluoride (HF) [1,2,3]. Fluorides enter the atmosphere during volcanic eruptions and from anthropogenic sources related to the development of industry and civilization, i.e., coal-fired power plants, steel and aluminum works, glass factories, brickyards and enamel factories, and plant-producing phosphorus fertilizers. Rotationally from the atmosphere, they return to the ground along with dust, snow, rain, or fog, and they can seep into ground and surface waters, as well as accumulate in soil and plants [1,4,5]. Plant protection agents and phosphorus fertilizers additionally enrich the soil with fluorides. Its level in nature determines the nature of the parent rock, chemical composition and pH, and ranges from several dozen to several hundred parts per million (ppm) [1,6]. The exceptions are the areas of endemic fluorosis, where this value can be exceeded many times over 8000 ppm [1,2]. Recently, there has been a disturbingly high increase in fluoride levels in the superficial soil layers. The reason is probably the widespread use of phosphorus fertilizers and the contamination associated with the emission of these compounds into the atmosphere [1].

Fluoride is also classified as a micronutrient, and in mammals it is present in the amount of 500 mg/kg DM (dry weight) [2]. It is related to the structure of the supporting tissue, and the hard tissues of the teeth, skin and hair. Taken up by bone tissue, it can be released therefrom, especially from the superficial layers based on iso and heteroionic exchange. As an element with high biological activity, it influences a number of processes taking place in living organisms. Fluorine ions are inhibitors or, less frequently, activators of many enzymes, and they influence the process of protein biosynthesis and carbohydrate and lipid transformations, thus modifying some biological functions of living organisms [1,3,5,7]. Fluoride has a strong antibacterial effect due to its inhibition of many enzymes, the most important of which are: enolase, F-ATPase, sulfatase, catalase, phosphatase, phosphoglucomutase, and others. In the case of bacterial enolase, fluorine is likely to combine with the magnesium found in the enzyme, thereby reducing glucose transport into the bacterial cell [8]. Another property is the exchange of the hydroxyl ion with the fluorine ion, thanks to which fluorapatite is formed, which is more resistant to the acidic environment than hydroxyapatite and also facilitates the remineralization of tooth tissues, especially enamel and dentine. Combined, these properties determine the meaning of their supply in order to prevent caries [9].

**Figure 1:**
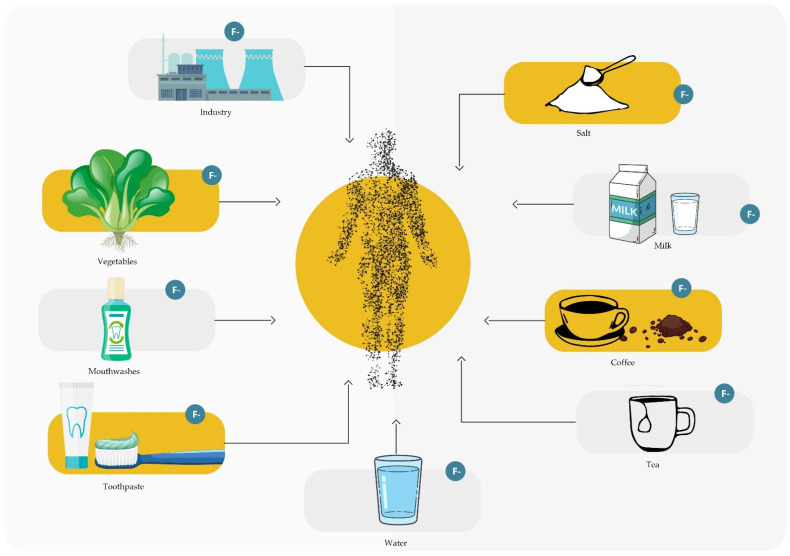
Fluoride supply sources.

Fluoride is an element that is taken up by the human body either with food or the air we breathe. In exceptional cases, it can penetrate the body through the skin layers. Absorption by airways occurs mainly in the presence of high concentrations of fluoride in the form of HF occurring in occupational exposure cases. In turn, the absorption of fluoride compounds in the gastrointestinal tract begins in the oral cavity (about 1%), in the stomach (40–50%), and then in the small intestine (20–30%). It was found that after an hour, it reaches the maximum concentration in the blood, and then it is mainly absorbed in the body by calcified tissues (99%), in addition to a small (about 1%) amount of soft tissues, i.e., kidneys, lungs, and brain. After 24 h from the consumption of fluoride, about 50% is excreted from the body, mainly in the urine but also in feces, sweat, saliva, and milk [1,2,3,5,7]. Figure 1 shows the potential sources of fluoride in the human body.

It is worth emphasizing that fluoride is commonly used among dentists to prevent and stop caries that have already occurred. Basic prophylaxis includes brushing your teeth at least twice daily with a toothpaste with an appropriate dose of fluoride, flossing the interdental spaces with fluoride-containing floss, or using rinses containing fluorine. Professional fluoride prophylaxis applies to people with an increased risk of caries, and includes the application of varnishes, gels, or foams by trained medical personnel. In the case of primary and secondary prophylaxis, the most important thing is to follow the rules regularly. Fluoride-containing filling materials and fissure sealants are also used to prevent caries. The formation of caries is associated with many factors, and the proper supply of fluoride is an important factor (but not the only one); therefore, the risk of occurrence should be individually determined by a specialist [10,11].

The aim of the article is to collect information on the properties of fluoride and the potential sources of its occurrence. The work focused on groceries, agents used in dentistry, and fluoride in the environment. The collected information will make it possible to reassure healthcare professionals of the importance of fluoride use in practice and to select its appropriate dose.

The data in the article were collected by many specialists in various fields of science. The latest and most widely recognized knowledge has been taken from papers published by international institutions, current medical books, and guidelines.

## 2. Cytotoxicity of Fluoride

### 2.1. Acute Poisoning with Fluoride Compounds

Fluorine compounds supplied to the body have beneficial biological effects, as long as they are not supplied in excess. Their supply in an unintended or uncontrolled manner, taking into account both the metabolism of fluoride, the chain of its transformations in the environment, as well as its increasing importance in industry and economy, may cause its accumulation in the body and the occurrence of side effects [12,13].

Most acute poisoning with fluoride compounds is caused by accidental ingestion. The most common fluoride compounds are sodium fluoride, sodium fluoroacetone, ammonium hydrofluoride, sulfuryl fluoride, hydrofluoric acid, and fluorosilicates. One example of mass poisoning was the 1943 report by Lidbeck, which documented the consumption of cockroach poison, which was added to scrambled eggs instead of powdered milk at Oregon State Hospital. Out of the 263 poisoned people, as many as 47 died. In the 1990s, as many as 296 people were poisoned in Alaska with fluoridated drinking water with a fluoride content of 150 ppm. The literature also describes several cases of deaths in children as a result of the consumption of fluoride-containing agents used as part of caries prophylaxis [3,4,5,14]. The toxic dose causing the early symptoms of poisoning is 1 mgF/kg body weight, the probably toxic dose is 5 mgF/kg body weight, and the safely tolerated dose is estimated at 8 mgF/kg. Regarding fluoride in dental products, Whitford’s safety considerations estimate the lethal dose as 14–28 mgF/kg body weight, while a certain lethal dose is defined at 32–64 mgF/kg body weight in adults and 15 mg F/kg body weight in children [15,16,17,18]. Table 1 presents the degree of fluoride poisoning and its dose.

Some authors argue that as little as 5 mgF/kg of body weight can cause a serious threat to life requiring immediate treatment [3,4,5,7]. At the same time, it was found that human susceptibility to fluoride poisoning increases in dry and hot climates, due to higher water intake and thus a higher supply of fluoride [1,2,19,20]. The pathomechanism of the toxic effect of fluorides is based on the formation of insoluble calcium fluoride in the body, which causes a significant decrease in blood calcium levels, i.e., hypocalcemia, and a simultaneous increase in potassium levels [3,4,5,16]. The symptoms of acute poisoning depend on several factors, such as the dose, the pH of the substance, the method of administration, the patient’s age, acid–base balance, and the degree of absorption. They usually appear shortly after the poison is ingested. There are four groups of disorders in this type of poisoning: 1. inhibition of enzymatic processes; 2. production of calcium complexes; 3. Shock; and 4. organ damage. Patients initially experience nausea and vomiting (they may even be bloody), followed by severe, diffuse abdominal pain and diarrhea. Additionally, there may be some non-specific symptoms, such as salivation, lacrimation, nasal discharge, weakness, cyanosis, damp and cold skin, shortness of breath, and headaches. The developing hypocalcemia leads to severe limb cramps and even tetany convulsions, arrhythmias, ventricular fibrillation, and disturbances in hemostasis leading to mucosal bleeding. An increase in plasma potassium levels, in turn, impairs functioning at the cellular level. As the poisoning progresses, the symptoms worsen. There is a drop in blood pressure, and arrhythmia, as well as respiratory and metabolic acidosis, appear, resulting in respiratory and cardiac arrest. If fluoride compounds come into contact with the skin or mucosa, chemical burns appear, such as deep, slowly healing, bleeding and sometimes abscessing lesions, ulcerations, or necrotic foci [1,3,4,5,7,15,16,17]. Gaseous fluoride (over 0.05 mgF/m^3^) after getting into the respiratory tract may cause coughing, dyspnea, chills, pathological respiratory murmurs, and even pulmonary edema [21].

### 2.2. Chronic Poisoning with Fluoride Compounds

Chronic fluoride poisoning occurs as a result of the long-term and continuous exposure of the body to fluoride compounds. The reason may be high concentrations of fluoride in drinking water, an excessive consumption of them from sources other than water, the not fully developed swallowing reflex in preschool children, use of supplementation, environmental contamination, and occupational exposure, as well as calcium deficiency, malnutrition, and disorders of the acid–base balance. Today, however, environmental pollution is an increasingly common cause of fluoride poisoning as industry develops and industrialization increases. The effects of such poisoning depend on the age at the time of exposure, duration of exposure, dose, kidney condition, and diet [1,3,7,15,17,19,22,23,24,25,26].

**Figure 2:**
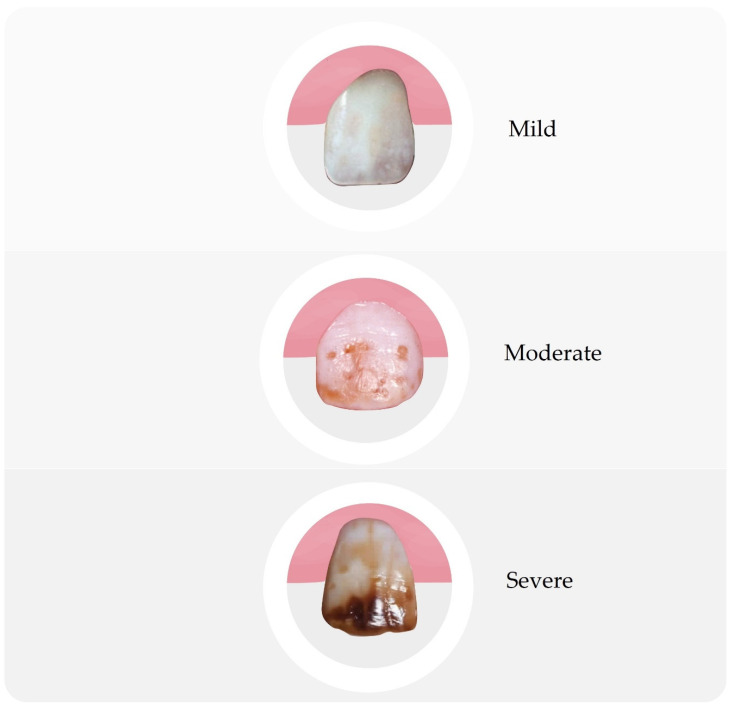
A scheme of different stages of fluorosis.

Dental fluorosis is the best-known type of systemic disorder caused by excessive fluoride intake. It is defined as a pathological condition resulting from excessive exposure to fluoride compounds during the development of enamel. Disease can result from exposure to either a large single dose or to smaller multiple doses, or it can be the result of continuous exposure to low levels of these compounds that disturb natural detoxification mechanisms [1,4,14,15,16,17]. Figure 2 shows various changes in teeth caused by too much fluoride intake.

The degree of advancement depends on the amount of fluoride compounds, their concentration, and duration of exposure, but also on the degree of development of tooth buds, individual susceptibility, and environmental factors [22,27]. The critical period for the entire dentition is the period between 11 months and 7 years of age, where from 15 to 30 months it is the cause of fluorosis of the permanent front teeth and first molars, and of the remaining teeth up to the age of 7. The pathomechanism of this disorder is not fully known, but most likely the processes accompanying biomineralization are affected, regardless of the origin of the tissue, resulting in disturbances in mineralization [7,15,22,27]. It manifests itself as the clouding, hypomineralization, and increased porosity of the enamel, and it can also lead to severe enamel hypoplasia. Clinically, enamel fluorosis occurs in the form of stains and discoloration of the enamel of varying intensity. Mild symptoms include thin, white stripes running under the enamel surface, visible after drying the examined tooth. More severe forms of the disease are characterized by small, irregular, whitish areas on the tooth surface, which are visible even without drying, followed by white but well-defined areas. In more advanced cases, the lesions forming the image of chalky teeth merge with the damage of the enamel surface in the form of pits. Severe stages of fluorosis result in the formation of pits in the enamel, which can form horizontal rims and discoloration. In the most severe form of the disease, the surface layer of the enamel is almost completely lost and the subsurface layer is exposed, producing brown discoloration, morphological changes, and the phenomenon of so-called enamel fragility, which occurs in places particularly exposed to chewing forces, i.e., on the incisal edges in the anterior teeth and on the tooth cusps in the lateral teeth [1,4,15,17,20,22,27]. In turn, damaged areas show an increased susceptibility to caries, which is confirmed by studies conducted among the population living in areas with an increased concentration of fluorine compounds [1,5,22,26]. The most widespread and most frequently used indicator for determining the severity of dental fluorosis is the Dean index, which describes the degrees of the disease (Table 2) [28].

Due to the pathomechanism of fluorosis, the changes occur symmetrically, while the degree of advancement in non-homologous teeth is different. Until recently, dental fluorosis was only endemic worldwide in areas with excessive levels of fluoride in the water. Recently, however, studies have confirmed the presence of fluorosis in regions with optimal, or even trace, fluoride concentration in water. This is evidenced by the fact that the formation of fluorosis is influenced by the total dose of fluoride taken from all possible sources, i.e., both from drinking water, air, food, and through various prophylactic and therapeutic methods that are currently very common [1,4,5].

Excessive exposure to fluorine compounds, especially from anthropogenic sources, i.e., industrial and occupational exposures, may also lead to the accumulation of this element in the bone tissue. The studies by Gabuda et al. [29] showed that after a week of exposing rats to a high concentration of fluoride, its accumulation in the bones was very high. Long-term exposure may lead to bone fluorosis. It is estimated that this applies to a situation where the daily amount of absorbed fluoride ranges from 10 to 20 mg and lasts for a period of 10–20 years. It can occur in several forms and lead to changes, both histopathological and morphometric, including an increase in bone density and the formation of exostoses. The changes and disturbances in the bone structure visible on X-rays are the thickening of the trabecular structure, which gives the impression of a rough, fuzzy, and cystic, milky color. The next stage is stiffness of the joints caused by calcification of the ligaments.

Currently, increasing attention is paid to the effect of overexposure to fluoride compounds on soft tissues. Numerous studies carried out on rats and rabbits prove the formation of morphological and biochemical changes on this medium, causing damage to the heart muscle, kidneys, liver, and brain [30,31,32,33,34,35,36,37,38,39,40]. Some authors describe cases of the influence of an excess of fluorine compounds on a reduction in IQ in children [35], the development of carcinogenesis, disturbances of reproductive and immune processes, and abnormalities in thyroxine synthesis, as well as a decrease in iodine uptake [40].

## 3. Sources of Fluoride

### 3.1. Groceries

The daily requirement of the human body for fluorine compounds depends on age. According to Moody (8), it should not exceed an average of 1.2 mg/day in children, 4.2 mg/day in adult men, and 3.6 mg/day in women. On the other hand, Olczak-Kowalczyk et al. determined the daily requirement of organisms, ranging from 0.01 mg for infants to 3.0 mg for adults (Table 3) [15].

It is now assumed that the optimal daily dose of fluoride for an adult with normal weight is on average 0.05 mg F/kg body weight, i.e., about 1.5–3.0 mg per day [14,41]. Research conducted in England and in European countries in 2014 showed significant differences in the average amount of fluoride supply. For adults, it ranged from 0.13 mg/day to 8.4 mg/day depending on age, sex, and country of residence [41]. One way of supplying this element is through food, which provides about 0.2–0.5 mg of fluoride. The amount of fluorine compounds in food products is small and ranges from 0.2 to 0.3 mg/kg of the product. Foods containing naturally significant amounts are sea fish and seafood (about 1.9 mgF/kg); tea (3.85 mgF/L); artificially fortified foods, including meat (82 micrograms/100g); sweets (40 micrograms/100g); and dairy products (50 micrograms/100g) [1,14,41]. It should also be remembered that vegetables and fruit have a great tendency to accumulate compounds of this element, both from the soil and from the air. Especially leafy vegetables, such as parsley, celery, beets, lettuce, spinach, leeks, and chives are a group of very active fluorine reservoirs. It is believed that due to the very easy absorption of even the smallest amounts of fluoride by plants, fruit and vegetables should not be grown in the immediate vicinity of the sources of fluoride emissions. Large amounts of fluoride are also found in canned foods. Another issue is the way food is prepared for consumption, including its processing and added ingredients, which ultimately also have a significant impact on the final fluoride content in the supplied food [1,2,42]. Cantoral et al. paid attention to the amount of fluoride in food, which depends mainly on salt, water, and the packaging utilized in the production process. In Mexico, salt contains 200–400 mg/kg, while water contains up to 4.5 mg/L. The authors pointed out that if you eat large amounts of food that are not essential to proper nutrition, which is a problem not only in Mexico, you may run the risk of having too much exposure to fluoride [43].

### 3.2. Water and Drinks

Water and drinks are an important source of fluoride. The natural source of fluorine compounds in water is the surrounding environment, i.e., the soil and the atmosphere. Fluoride is found in all natural waters in a certain concentration. Sea water usually contains about 1 mg/L, while rivers and lakes contain less than 0.5 mg/L. In underground sources, fluoride concentrations can range from low to high depending on the type of rock. The concentration of fluoride is limited by the solubility of fluorspar in water, which means that in the presence of 40 mg Ca/L it should not exceed 3.1 mg F/L [1,44,45]. Waters with a high concentration of fluorine occur in geographical zones associated with the presence of: (a) marine sediments in mountain areas, (b) volcanic rocks, and (c) granite and gneiss rocks. The optimal level of fluoride in drinking water is around 0.7 mg F/L (0.7 ppm) [46]. Waters containing less than 1.0 mgF/L are treated as drinking waters, while those above 5 mgF/L are treated as medicinal waters [1,2]. The correct amount of water recommended in nutrition is on average 2 L per day, which means a supply of about 0.2–2.4 mg of fluoride to the body, assuming the optimal level of this element in water [47]. An example of places in the world where the natural concentration of fluorides in water is significantly higher than the safe and optimal and exceeds 5 mg/L are: Algeria, Thailand, Mexico, Africa [19,22,26,44,48]. In some regions of China and India, due to the natural supersaturation of geological deposits with fluorine compounds, the level of fluoride reaches even 10–40 mg/L [23,24]. It poses an additional threat in poor countries, where the only source of drinking water is rainwater—its composition is impossible to control, and contamination of the area with fluoride translates into its content in the water [26]. Dean’s research [25] in the 1950s showed a direct relationship between an increase in the concentration of fluoride in drinking water above 1 ppm and an increased frequency of fluorosis. Therefore, such a situation poses a high risk of accumulation of fluoride in the body and requires the removal of the excess of these compounds from the water. In the case of children of preschool and school age, very important sources of fluoride include juices, fruit drinks, and carbonated drinks [1].

In the case of bottled water, the fluorine content usually does not exceed 0.6 mg/L. Therefore, in the case of caries prevention, an additional supply of fluoride is needed. On the other hand, in the case of too much fluoride content in the consumed water, bottled water can be an alternative way to reduce the supply of fluoride to the body [49,50,51].

Fluoride is also found in coffee. Wolska et al. showed that depending on the method of brewing and the type of coffee (robusta, arabica, green, etc.), the drink contained 0.013–0.502 mg/L. The highest content is in green coffee, which is not heat-treated. Robusta and arabica are the most popular coffee varieties consumed in the world, with arabica being more common [52,53].

Tea, similar to coffee, is a frequently consumed drink worldwide. It is an infusion made from the leaves of Camellia sinensis, and it varies in fluoride content depending on the country, region, year, and method of tea processing. There are also many tea-based drinks on sale. Peng et al. in their studies indicated very large differences in the content of fluorine, amounting to 8.49–803.94 mg/kg. The authors drew attention to the fact that drinking tea does not in any case cause too much fluoride intake in adults [54]. The Indian study focused on the age, quality of tea leaves, and the use of fertilizers and pesticides. Older leaves contain more fluoride than younger leaves, similarly from market tea samples, which are a lower-quality product than loose tea. Both children and adults had too much fluoride in the case of drinking tea from old leaves and market tea samples. The authors recommended avoiding these teas in areas where there is a large amount of fluoride in the water, replacing them with other infusions, e.g., mint, rooibos [55]. Scientists from Taiwan had similar conclusions, pointing to the high content of fluoride in teas, which, particularly in combination with the high content of this element in water, can cause side effects. Some studies indicated a lowered IQ in children or increased bone fragility due to fluorosis [56]. Additionally, bottled tea drinks contain too much fluoride. In Taiwan it is even 25.7 mg/L, while in Germany it is a maximum of 1.79 mg/L. M. Whyte tested beverages in the US with a maximum of 4.2 mg/L, with 11 samples exceeding the maximum content of 2.4 mg/L. Bone fluorosis is a disease more common in tea-drinking countries due to the long-term supply of too much fluoride [57]. Similar results and conclusions were obtained by Rirattanapong et al., with 2/3 of the products they tested exceeding the recommended dose of fluoride. The results of individual products varied significantly; however, the authors wanted to point out the lack of regulation in the case of Thailand, which is of particular importance in the case of children’s fluorosis [58]. Studies have shown that the longer the tea is brewed, the greater the concentration of substances (fluoride, vitamins B1, B2, PP, C). It should be emphasized that exceeding safe doses of fluoride concerns people who drink a lot of tea. Malinowska et al. determined that in the case of 1 L of consumed drink, the dose is most exceeded in the case of black tea, up to 3 times, and in the case of green and white tea, the dose is maximally higher by 122% and 112%, respectively. However, the scope, as in other studies, differed significantly depending on the tested sample [59,60]. There is no doubt that tea is a significant source of fluoride in the diet, and tea consumption should be taken into account when selecting appropriate supplementation [61].

In the case of popular drinks, such as Coca Cola, Pepsi, Mirinda, Fanta, and Sprite, in the case of Ethiopia, scientists compared their results to similar studies from around the world. Fluoride content varied depending on the type and packaging (glass or plastic), ranging from 0.03 to 0.23 mg/L for glass bottles and 0.05 to 0.27 mg/L for plastic bottles. For this reason, there is little risk of obtaining too much fluoride from consuming these foods [62].

### 3.3. Drugs and Agents Used in Dentistry

From the point of view of dentistry, the role that fluoride compounds play in the development of hard tooth tissues and how they can inhibit caries is important. For over 50 years, fluoride compounds have played an important role in the prevention of tooth decay. We are talking about two mechanisms of action of fluorides: pre-eruption and post-eruption. The pre-eruption action before tooth eruption is based on the primary mineralization of the tooth’s organic matrix and the pre-eruption enamel maturation. They play the role of a catalyst in the formation of hydroxyapatites, promote the formation of the correct crystal network of enamel, participate in the process of removing water and organic substances from the newly formed enamel, and make the enamel of developing tooth buds less susceptible to demineralization. Moreover, they influence the more favorable morphology of the teeth, which facilitates the processes of self-cleaning of fissures on the chewing surfaces. The post-eruption effect of fluoride is based on its influence on the adhesion of bacterial plaque and bacterial metabolism, inhibiting the demineralization process and supporting remineralization [1,2,15,16,17,63]. The reduced adhesion is probably related to the increase in pellicle thickness [64].

Fluoride prophylaxis methods can be divided into endogenous and exogenous, as well as individual and collective. The latter, however, are non-specific methods, enabling the same procedure to be applied to all members of a given population, regardless of the level of risk of caries.

### 3.4. Endogenous Fluoridation

Endogenous methods of fluoride prophylaxis are based on the supply of a certain amount of this substance to the body, depending on the demand and age. These include the fluoridation of drinking water, use of tablets or drops with fluoride, and fluoridation of foodstuffs, such as salt and milk.

The fluoridation of water was introduced in Grand Rapids in the United States in 1945, then in Canada, and a few years later in 1952 in Europe. In the 1970s, this method of caries prevention spread virtually all over the world, including the Far East, Australia, and New Zealand, comprising over 120 million people. Currently, fluoridated water is used by 75% of the US population and 83% of the Australian population, which means over 200 million people [1,14,16,44,45,48]. The optimal and completely safe concentration of fluoride in water is 0.5–1.0 mg F/L. Based on a UK Public Health report from 2014 [41], a significant decrease in the incidence of tooth decay in areas with fluoridated water was found: in a group of 5-year-olds by 28% and in a group of 12-year-olds by 21%. Partial withdrawal from this type of prevention was caused by the so-called failure to adjust the supply of fluoride to individual needs and the increased risk of an uncontrolled supply of this element from anthropogenic sources as a result of industrialization and environmental contamination [1,16,17,44,47]. In the case of the fluoridation of drinking water, the so-called the “halo” effect consisted of the indirect impact of the application of this method on the areas not covered by the prevention. This effect is based on the use of drinking water artificially enriched with fluoride for the preparation of foods and beverages exported to other countries where fluoridation is not carried out or is not required. This may contribute to significantly higher fluoride uptake by communities—data that is difficult to control and monitor [1,2].

The fluoridation of foodstuffs mainly concerns table salt and milk. In the case of table salt, this method was first used in Switzerland in the 1940s by Doctor Wespi. This method was popularized in France, Hungary, Cuba, and Mexico, as well as in Germany. According to the National Research Council Report [47], it is considered a possible alternative to fluoridating water in areas where it cannot be prevented or where the natural content of fluoride in the water is low. The optimal amount of this element for domestic salt is 400 mg/kg, and for salt used in a wide range, e.g., in bakeries, canteens, etc., the optimal amount is 200 mg/kg [1,14,16,17,41].

The fluoridation of milk is very difficult and costly, and its use is very limited due to the difficulties of consistently delivering it to the body. This method was first used in the mid-1950s in children’s drinks. Currently, this type of prophylaxis is quite sporadic, mainly in schools as part of preventive measures, where milk with a content of 5 mgF/L is given for 200 days [1,14,16,17].

Supplementation with fluoride tablets or drops was introduced in the 1940s. Wherever water fluoridation was impossible to introduce, and the natural content of these compounds remained below the optimal level, fluoride drops or tablets were used. In this type of supplementation, it is necessary to accurately determine the right dose because there are studies showing the relationship between the frequency of fluorosis and tablet supplementation. The dosage depends on the patient’s age and the level of fluoride in the water, and it requires systematic supply for many years [1,2,14,15,16,17]. The models of caries pill prophylaxis vary from country to country. The figures below provide recommendations for tablet fluoride supplementation depending on the level of fluoride in water, established by the European Academy of Pediatric Dentistry (EAPD) in 2009 (Table 3) [14] and the American Academy of Pediatric Dentistry (AAPD) in 2018 [65] (Table 4).

Fluoride tablets or drops are used for 200–250 days a year, with a break in the summer. Young children are given drops or tablets dissolved in water, and older children are given a tablet orally, with slow sucking without chewing in order to maximize the beneficial local effect [15,17]. Systemic fluoride supply is effective only when it is used regularly and in accordance with guidelines in the pre-eruption period of dentition development; that is, between the ages of two and eight. After this time, systemic fluoride intake has no effect on the dentition [1,17]. In the case of this type of prophylaxis, the risk of overdose is mainly due to incorrect dosing, as well as the possibility of uncontrolled supply of fluoride from other sources [1]. Currently, due to high contamination with this element of the environment, tablets and drops should be recommended with special care, taking into account individual needs, environmental conditions, and other possible routes of fluoride supply. It is currently known that the content of incorporated fluoride in enamel does not significantly reduce the risk of caries, while an excessive and difficult to monitor endogenous supply may cause dental fluorosis [15,17].

### 3.5. Exogenous Fluoridation

Contact fluoridation methods rely on the direct action of fluoride compounds on the tooth surface. They were introduced as a group prophylaxis in the early 1940s [1]. These can be treatments such as rubbing, brushing, rinsing, flossing, or compressing with the use of both organic compounds (aminofluorides) and inorganic compounds (sodium fluoride, tin fluoride, acidified sodium monofluorophosphate) of various concentrations. In the case of professional procedures performed in offices under the supervision of a doctor, the following are used: 2% and 5% neutral sodium fluoride, 1.1% sodium fluoride, 4–10% tin fluoride, 2% acidified sodium monofluorophosphate, and amine fluorides. For home treatments, toothpastes and rinses with an average concentration of 10 times lower are used, which ensures safety of use [14,15,16,17,63,66,67]. However, it should be remembered that this type of procedure may also cause the accumulation of fluoride in the body. This is due to the ingestion, especially of children, of toothpaste or mouthwash, the use of preventive measures not adapted to a certain age, the use of too many preparations, or a lack of control on the part of parents and guardians [1,5,15,17,66,67]. Pastes or gels with an increased content of fluoride (1.1%) accidentally eaten may cause acute poisoning with a fatal outcome [15,66].

### 3.6. Fluoride Mouth Rinse

Fluoride’s remineralizing and cariostatic properties have been a major advantage in fluoride-enhanced mouth rinses [68]. Their general use is currently advised in cases of patients suffering from active carious lesions, gingival recessions, and chemo- or radiotherapy. They can also act as an auxiliary method in preventing restorative treatment in generally healthy patients that are at higher risk of caries due to orthodontic appliances or prostheses [69]. Although fluoride in a mouth rinse acts in the oral cavity for a short time, it is still considered to have a positive effect on the prevention of caries [70]. As Carvalho et al. [71] pointed out, the use of toothpastes together with mouth rinses reduces bacterial caries activity with more intensity than the use of either of them alone. The use of fluoride mouthwash, as well as fluoridated toothpaste, has been reported to have a significant effect on decreasing dental caries [72]. Aminabadi et al. [73] proved that use of fluoride mouthwash appears to be effective both in individuals and large groups. In the case of cleaning the tooth’s surface, it is worth paying attention to the presence of non-humus tissue defects, which may be caused by an incorrect brushing technique or diet [17,74]. 

Typically, 0.05% and 0.2% sodium fluoride mouthwashes are available. It is important to underline that according to health standards, 0.05% mouthwash is a weak solution and needs to be applied daily, while the stronger 0.2% should be used once a week. Mouth rinse applications should be intended for patients of 6 years and older [75]. Although it has a plethora of advantages, such as low price, convenience in use, and easy application, it has to be carefully administered and strictly controlled, especially in children [76], in order to avoid overdose.

### 3.7. Fluoride Varnish

Fluoride varnish is a natural material that is composed of tree resin enriched with a concentrated fluoride. As Miller et al. [77] pointed out, its features allow for persistent adherence to the tooth surface, which leads to the prolonged uptake of fluoride by enamel. In dentistry, fluoride varnish acts as one of the several sources of additional fluoride administration. It is developed in order to prolong the contact between the tooth surface and the fluoride, reinforcing the tooth structure, making it less susceptible to carious lesions [78]. Its application ensures a longer duration of action of fluoride on the tooth when compared with fluoride mouth rinses. The reason for the topical application of fluoride is to eliminate tooth decay or to limit its current development. Fluoride varnishes are widely used in dentistry as a preventive measure, even with infants [79]. The active ingredient that is most common in fluoride varnishes is a 2.26% fluoride from a suspension of 5% sodium fluoride (NaF) [80]. Duraphat was the first commercially available product containing fluoride varnish [78]. Among others, the ones that are common include Duraflor, Fluor Protector, or Cavity Shield. There are several studies confirming the effectiveness of Duraphat in cases of carious lesions in pedodontic patients [81,82]. It is important to underline that recent studies [83,84] confirmed the superiority of fluoride varnishes enriched with calcium compounds, including compounds, such as casein phosphopeptide amorphous calcium phosphate (CPP-ACP) and phosphate tri-calcium (TCP). CPP-ACP acts as a neutralizer to acidic pH values, enhances the remineralization of incipient carious lesions by hydrolyzing hydroxyapatites, and inhibits bacterial growth. TCP on the other hand resembles a hydroxyapatite structure and reduces the chelation of phosphate, fluoride, and calcium ions on dental surfaces. When it comes to general methods of fluoride varnish application, there are a few important steps. Primarily, the procedure should begin with the removal of excessive moisture from the teeth surface using, e.g., an air syringe. About 1 mL should be enough for the whole dentition [85]. The varnish should be applied as a thin layer on the teeth surface, while no additional drying is required after the procedure. The stage after applying the varnish is also very important. On the same day, the patient should eat only soft food and refrain from brushing the teeth on the same day after applying the varnish in order to extend the time of contact between the varnish and the tooth surface.

### 3.8. Fluoride Foam and Gel

Fluoride foams have a similar concentration of fluoride, as well as a similar pH when compared to 1.23% F acidulated phosphate gels (APF) [86,87]. APFs are currently one of the most popular compounds of fluoride; however, it is important to underline that both foams and gels are inferior in the case of the efficacy of fluoride release when compared with varnishes [88]. A method of fluoride foams and gels application is the insertion of the material on a special trimmed tray, with the patient being asked to keep the appliance in his mouth for 4 min while leaning forward [89,90]. Both types of materials have been commonly used in caries prevention programs worldwide [91,92,93]. According to Twetman et al. [75], the foam material only requires a fifth of the weight of the gel to completely cover the teeth; however, the clinical evidence regarding foams has confirmed that their efficacy is lower than fluoride gels [63]. Fluoride foams on the other hand cause a lower uptake of fluoride ingestion following their application compared with gels [94].

### 3.9. Filling Materials

**Figure 3:**
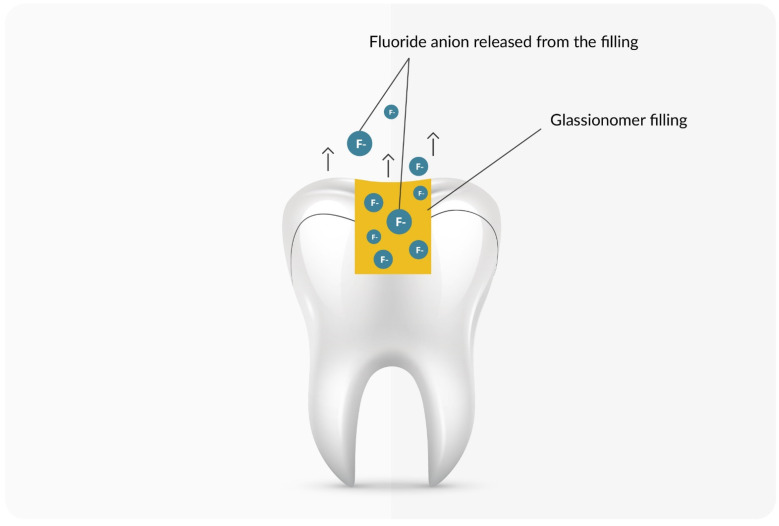
A scheme of a tooth with a filling containing fluoride ions that are released in the oral cavity.

Fluoride-releasing materials mainly include glass ionomer cements, compomers, and modified composites. Depending on the type of material, the amount of fluoride released is different. It positively influences the microleakage problem in dental fillings by reducing the amount of secondary caries [95,96,97]. In addition to its use in restorative dentistry, Nascimento et al. noted the use of some resins and compomers in reducing the occurrence of white spots in orthodontic brackets. However, due to insufficient evidence, they do not consider the use of these materials an effective anti-decay agent in bracket cements [98]. Glass ionomers have about 5 times greater fluoride release than compomers and are about 21 times greater than modified resins. In the case of glass ionomers, it was about 480 µg/cm^2^, and one should pay attention to the burst effect, which is the increased release of fluoride into the oral cavity [98,99]. Glass ionomers are also enriched with nano-hydroxyapatite silica powder, which increases, among other things, ion exchange [100,101]. Figure 3 shows the release of fluoride in the oral cavity environment, taking into account the burst effect.

### 3.10. Fissure Sealants

Fissures and pits of the occlusal surfaces of teeth are more prone to caries due to their anatomical structure, which enhances dental plaque retention. The idea of fissure sealants is to form a physical barrier that stops the formation of the carious lesion [102]. They ensure a cariostatic effect for a long time [103,104]. It is worth paying attention to the positive aspect of the regular release of small amounts of fluoride ions, which have a significant impact on the prevention of caries [105,106].

### 3.11. Medicines and Agents Used in Medicine

Fluoride is a strong stimulator of bone tissue formation, influencing both the bone formation process and the increase in its density [27,107]. Thanks to this, it is used in the treatment of osteoporosis as a first-line drug (bisphosphonates—3rd generation). These drugs, when used continuously, provide 10–25 mg of NaF/day, which are potentially an important source of this element in the body [108,109]. Therefore, it is necessary to constantly monitor the level of fluoride in the environment and not combine this therapy with any other method of endogenous prophylaxis. Another way of using fluoride compounds in medicine is through diagnostic tests. As early as 1962, Blau used them for imaging the skeletal system. He exploited the rapid removal of fluoride from plasma and its high affinity to bone. Modern diagnostics use positron emission tomography (PET) with the use of fluoride, which enables the assessment of pathophysiological processes in the course of osteoporosis, as well as the evaluation of innovative methods of pharmacological treatment of this and other metabolic bone diseases [110,111].

### 3.12. Industry and Econoamy Development

With the development of civilization, industry, agriculture, and large urban agglomerations, the environmental factor seems to be more and more important in degradation. Fluorine and its compounds are important and very powerful environmental poisons. The main sources of fluoride emissions are coal-burning power plants, steel and aluminum works, glass factories, brick and enamels factories, and factories producing phosphorus fertilizers. They can shape the concentration of fluorine in the environment up to the level of 500 ppm [1,67]. In the process of the incomplete combustion of coal, oil, gas, and garbage, polycyclic aromatic hydrocarbons are produced, in the group of which there are such compounds as benzofluorants, fluorants, and fluorens. Benzofluorants have a strong carcinogenic effect through the formation of metabolites in the human body that initiate the development of the neoplastic process [112]. In areas where fluorine-containing fuel is used for smoking and where fluorine-containing fertilizers are produced and used in agriculture, the concentration of this element in the air is increased, which leads to increased exposure by inhalation. In China, where air pollution with fluoride is very high, more than 10 million people are affected by fluorosis [1,2,24,113]. In addition to environmental pollution as a result of industrial emissions, improperly managed production waste may also be a source. Particular care should be taken when cultivating plants and vegetables in such surroundings because the ease of assimilation of these compounds may lead to their significant accumulation. It is believed that the amount of fluoride in plants should not exceed 35–40 ppm [1,21]. At a higher level, adverse effects on the human body can be expected. This is especially important when the difference between the necessary safe and toxic doses is small. When assessing the amount of fluoride in plants in rural areas and urban agglomerations, it was found that in the latter it is much higher, which is related to a higher concentration of pollutants [1,114]. Additionally, the levels of fluoride are influenced by chemicals in agriculture and the widespread use of phosphorus fertilizers and insecticides, where the content of this element ranges on average from 8.5 to 38 mg/L [1,4,5,44].

## 4. Discussion

In recent years, there has been more and more information about the harmful effects of fluoride, an example of which is the relatively easy availability of fluoride-free toothpastes. However, this is not new to dentistry. Easy access to the internet, unfortunately, favors the spread of false information, which finds fertile ground under catchy slogans such as the “great lie” and “collusion of pharmaceutical companies”. Therefore, it is important to provide scientific evidence for the addition of fluoride [115,116].

On a larger scale, the fluoridation of water and the addition of fluoride in medical products began in the 1940s in countries at a higher level of development. However, when combined with naturally occurring fluoride in food, drink, and water, it can cause dental and bone fluorosis, which are irreversible diseases [117]. For this reason, the WHO places great emphasis on the control of the amount of fluoride consumed, which is primarily beneficial for the population, especially for children. In the case of caries, it is best to prevent it, and fluoride is an effective tool [118]. Primary teeth caries is observed in as many as 530 million cases worldwide. Undoubtedly, it is caused by many factors, however, properly supplementing and disseminating knowledge about fluoride is an important way to reduce this disease in the world [119].

In their extensive work, Iheozor-Ejiofor et al. collected information on the effects of fluorinated water and other fluoride-containing agents. The reduction in the amount of caries is 35% for milk teeth and 26% for permanent teeth in children. It is worth mentioning that dental caries in adults is strongly associated with inadequate hygiene, diet, and other factors. It should also be noted that the effectiveness of fluorinated water was greater in less developed countries, where access to fluoride toothpastes and other agents, as well as hygiene standards, are much worse than in developed countries [48]. Additionally, O’Mullane et al. indicated the beneficial effect of fluoride on oral health. They comprehensively presented the methods and programs of fluoridation around the world, and their conclusions only confirm the effectiveness of this method. In the dentist’s office, depending on the risk of caries, there are various agents that are used only by professionals as prophylactic agents. Additionally, dental materials that contain fluoride ions are often used in offices because of their properties that reduce the likelihood of secondary caries or, in cases of the inaccurate removal of caries, stop it. This is a good argument for the use of fluoridization for people who have any doubts about the harmfulness or effectiveness of this method [120].

On the other hand, there are many studies related to lowered IQ in children who lived in places with water with increased fluoride content. However, the authors themselves reported that they focused on the content of fluoride in the water, and they had no information about other sources of fluoride, such as dental preparations, food, or drinks. Therefore, they are unable to pinpoint the exact dose that has been consumed. It is worth noting that breastfed children did not receive an increased dose of fluoride with their milk, despite living in a region where the water contained too much fluoride. It should also be noted that in children with too-low or normal fluoride intake, no reduced IQ levels were observed [121,122,123,124,125]. That is why controlling fluoride consumption among children is so important; not all regions have naturally high amounts of fluoride in their water. However, we should be aware of the level of fluoride in the water and the presence of fluoride in many foods consumed daily. Tea is important as it can supply a large amount of fluoride due to cultural traditions of drinking and the use of tap water [126]. In the case of prenatal fluoride dosing, the tests were of insufficient quality; however, no sense of supplementation was found in them [127]. Table 5 shows different foods and, for comparison, some oral care products not intended for consumption. It should be noted that there are large differences in the content of fluoride in some products. The most common cause is differences in the countries where the research was carried out and their specific conditions. For this reason, it is important for fluoride prophylaxis to be selected by a professional who, thanks to dental preparations, is able to optimize the supply of fluoride to the body.

## 5. Conclusions

Currently, there is wide access to knowledge about fluoride, which unfortunately opens the door to the propagation of false information. Fluoride in optimal amounts is an element that has a positive effect on the functioning of living organisms. Correct dosing is well known and documented; therefore, we must remember when taking fluoride-containing products to consider the health consequences of overdosing by carefully monitoring its supply. The current position of experts is that the use of fluorine compounds in the broadly understood fluoride prophylaxis is a correct, safe, and scientifically proven procedure. In any case, the choice of the method and prophylactic measure should depend on the individual needs of the patient.

## Figures and Tables

**Table 1:** Table showing the relationship between fluoride dose and toxicity [17].

Toxicity of Fluoride		
1	Early symptoms of poisoning	1 mgF/kg body weight
2	The probably toxic dose	5 mgF/kg body weight
3	The lethal dose (LD)	14–28 mgF/kg body weight
4	The certain lethal dose (CLD)	32–64 mgF/kg body weight

**Table 2:** Classification of dental fluorosis by Dean’s index.

The Degree of Fluorosis on a Numerical Scale	The Degree of Fluorosis	Clinical Features
1	Normal	Flawless, glittering surface with various shades of white, ecru, cream
2	Questionable	It differs from the normal degree, with the presence of single white spots or fleck changes
3	Very Mild	Minor, intensely white regions with a lack of transparency on up to 25% of the surface, with no brown spots
4	Mild	The changes are similar to a lower degree, but cover up to 50% of the area
5	Moderate	There are already brown spots and the lesions cover most of the tooth surface, but the shape of the tooth usually does not change
6	Moderately Severe	Cavities are more frequent, and they are also deeper and more extensive
7	Severe	Visible hypoplasia in the form of comprehensive lesions and a color from brown to even black

**Table 3:** Human body daily requirements for fluoride compounds: optimal and acceptable.

Recommended Intake of Fluoride mg/day	0.01–0.7	0.5–0.9	0.7–1.3	1.0–2.2	2.0–2.8	3.0–3.6
Age	0–6 months	6–12 months	1–3 years	4–8 years	9–13 years	14–18 years

**Table 4:** Comparison of recommendations for endogenous fluoride supplementation by EAPD and AAPD.

Recommendations Developed by	Age	Concentration of Fluoride in Drinking Water		
		<0.3 ppm	0.3 ppm–0.6 ppm	>0.6 ppm
EAPD	0–24 months	0 mg	0 mg	0 mg
	2–6 years	0.25 mg (0–3 years)	0.25 mg (3–6 years)	0 mg
	7–18 years	0.5 mg	0.25 mg	0 mg
AAPD	0–6 months	0 mg	0 mg	0 mg
	6 months–3 years	0.25 mg	0 mg	0 mg
	3–6 years	0.5 mg	0.25 mg	0 mg
	6–16 years	1.0 mg	0.5 mg	0 mg

**Table 5:** A table showing different sources of fluoride consumed or used in the human mouth.

Selected Sources of Fluoride in the Human Body			
Fluoride Source	Estimated Average Fluoride Content	Method of Product Administration	References
Fresh water	0.5–40 mg F/L	Beverage	[24,25,48]
Salt	100–400 mg F/kg	Food	[1,43,126]
Fluoridated water	0.5–1 mg F/L	Beverage	[1,2]
Sea food	1.9 mg F/kg	Food	[1,14,41]
Meat	820 µg F/ kg	Food	[1,14,41]
Coffee	0.013–0.502 mg F/L	Beverage	[53,54]
Tea, bottled tea	1.79–803.94 mg/L	Beverage	[55,58]
Carbonated drinks	0.03–0.27 mg F/L	Beverage	[63]
Fluoridated milk	5 mg F/L	Beverage	[1,14,16,17]
Toothpaste for adults	1450–5000 mg F/kg	Dental agent	[127]
Fluoride varnish	22,600 mg F/L	Dental agent	[80]
Fluoride foam and gels	5000–12,500 mg F/kg	Dental agent	[11,17,75]
Fluoride rinses	225–900 mg F/L	Dental agent	[11,17,73]

## Data Availability

Not applicable.
